# Augmented peroxisomal ROS buffering capacity renders oxidative and thermal stress cross-tolerance in yeast

**DOI:** 10.1186/s12934-021-01623-1

**Published:** 2021-07-12

**Authors:** Nai-Xin Lin, Rui-Zhen He, Yan Xu, Xiao-Wei Yu

**Affiliations:** Key Laboratory of Industrial Biotechnology, School of Biotechnology, Ministry of Education, Jiangnan University, 214122 Wuxi, People’s Republic of China

**Keywords:** Yeast, *Komagataella phaffii*, Thermotolerance, Reactive oxygen species (ROS), Peroxisome, Catalase

## Abstract

**Background:**

Thermotolerant yeast has outstanding potential in industrial applications. *Komagataella phaffii* (*Pichia pastoris*) is a common cell factory for industrial production of heterologous proteins.

**Results:**

Herein, we obtained a thermotolerant *K. phaffii* mutant G14 by mutagenesis and adaptive evolution. G14 exhibited oxidative and thermal stress cross-tolerance and high heterologous protein production efficiency. The reactive oxygen species (ROS) level and lipid peroxidation in G14 were reduced compared to the parent. Oxidative stress response (OSR) and heat shock response (HSR) are two major responses to thermal stress, but the activation of them was different in G14 and its parent. Compared with the parent, G14 acquired the better performance owing to its stronger OSR. Peroxisomes, as the main cellular site for cellular ROS generation and detoxification, had larger volume in G14 than the parent. And, the peroxisomal catalase activity and expression level in G14 was also higher than that of the parent. Excitingly, the gene knockdown of *CAT* encoding peroxisomal catalase by dCas9 severely reduced the oxidative and thermal stress cross-tolerance of G14. These results suggested that the augmented OSR was responsible for the oxidative and thermal stress cross-tolerance of G14. Nevertheless, OSR was not strong enough to protect the parent from thermal stress, even when HSR was initiated. Therefore, the parent cannot recover, thereby inducing the autophagy pathway and resulting in severe cell death.

**Conclusions:**

Our findings indicate the importance of peroxisome and the significance of redox balance in thermotolerance of yeasts.

**Supplementary Information:**

The online version contains supplementary material available at 10.1186/s12934-021-01623-1.

## Background

*Komagataella phaffii* (formally known as *Pichia pastoris*) is a widely used platform for heterologous protein productions and has become a potential choice for metabolic engineering as well [1]. *K. phaffii* has barely been reported to grow at temperatures higher than 30 °C, and the fermentation efficiency decreases when the temperature is higher than 28 °C. However, during the fermentation process, the fermenter temperature easily rises, leading to reduced production efficiency [2]. In most industries, this overheating problem is partly overcome by using cold water to cool the fermenter walls which adds to production costs [3, 4]. The potential of thermotolerant yeast in industrial applications has been extensively appreciated and hence an exponential upsurge in relative research is still in progress [5]. The thermotolerance of the model yeast *Saccharomyces cerevisiae* has been deeply investigated [6]. Regrettably, research on a thermotolerant *K. phaffii* have barely been reported.

Cross-talk of protective mechanisms occurs in yeasts when responding to various environmental stresses. For instance, heat shock response (HSR) and oxidative stress response (OSR) are involved in cellular thermotolerance, and thermotolerant *S. cerevisiae* is often tolerant to oxidative stress [7, 8]. The functional role of transcription factors (TFs) in cells is clear, however, at least 11 stress-responsive TFs change simultaneously under oxidative or thermal stress in *S. cerevisiae* [9]. Oxidative stress from H_2_O_2_ can induce HSR in *S. cerevisiae*, and many other stresses such as acetic acid can also induce HSR by causing oxidative stress on *S. cerevisiae* [10]. Thermal stress simultaneously induces HSR and OSR under an aerobic environment [11, 12]. Although HSR and OSR have been substantially explored, their ordinated regulation mechanism is poorly understood. The cross-talk of protective mechanism still needs to be further explored.

Redox balance is associated with the oxidative stress tolerance and thermotolerance of yeast. Intracellular reactive oxygen species (ROS) accumulation is the main cause of damage under stress conditions. Eliminating these radicals can effectively improve the stress tolerance of *S. cerevisiae* [13, 14]. The antioxidant defence system including enzymatic and non-enzymatic parts [15] is the key to reducing cellular ROS levels. Overexpression of antioxidant enzymes improves the thermotolerance of *S. cerevisiae* during ethanol production [13, 16]. The secretion of glutathione protects the population of *S. cerevisiae* from adverse thermal stress [17].

In general, heat-induced damages are due to an imbalance of protein homeostasis [18], which is maintained by function factors and systems including heat shock proteins (HSPs) [19], trehalose [20], ubiquitin-proteasome system [21], and autophagy [22]. In particular, the increased levels of HSPs are the basis of heat stress resistance. HSPs function as molecular chaperones by either stabilizing new proteins to ensure their correct folding or refolding misfolded proteins to proper conformation [8, 23]. Trehalose, which is one of the compatible solutes synthesized during adverse environmental conditions, protects cells by holding proteins and membranes in their native conformation [24, 25]. Ubiquitin marks denatured proteins for recognition and degradation [26]. Overexpression of the ubiquitin ligase *RSP5* accelerates the ubiquitination of cell proteins and enables the superior thermotolerance of *S. cerevisiae* [27]. However, the thermotolerance mechanism of yeast is not comprehensive enough, and further research is still needed.

In this study, a thermotolerant *K. phaffii* G14 was acquired by simulating natural evolution in the laboratory. The physiological and biochemical characteristics of G14 were validated and compared. After transcriptome data analysis and result verification, a thermotolerance mechanism in G14 was unveiled. Our study revealed a novel mechanism of the thermotolerance of yeast.

## Results

### Development and validation of the thermotolerant *K. phaffii* strains

**Fig. 1:**
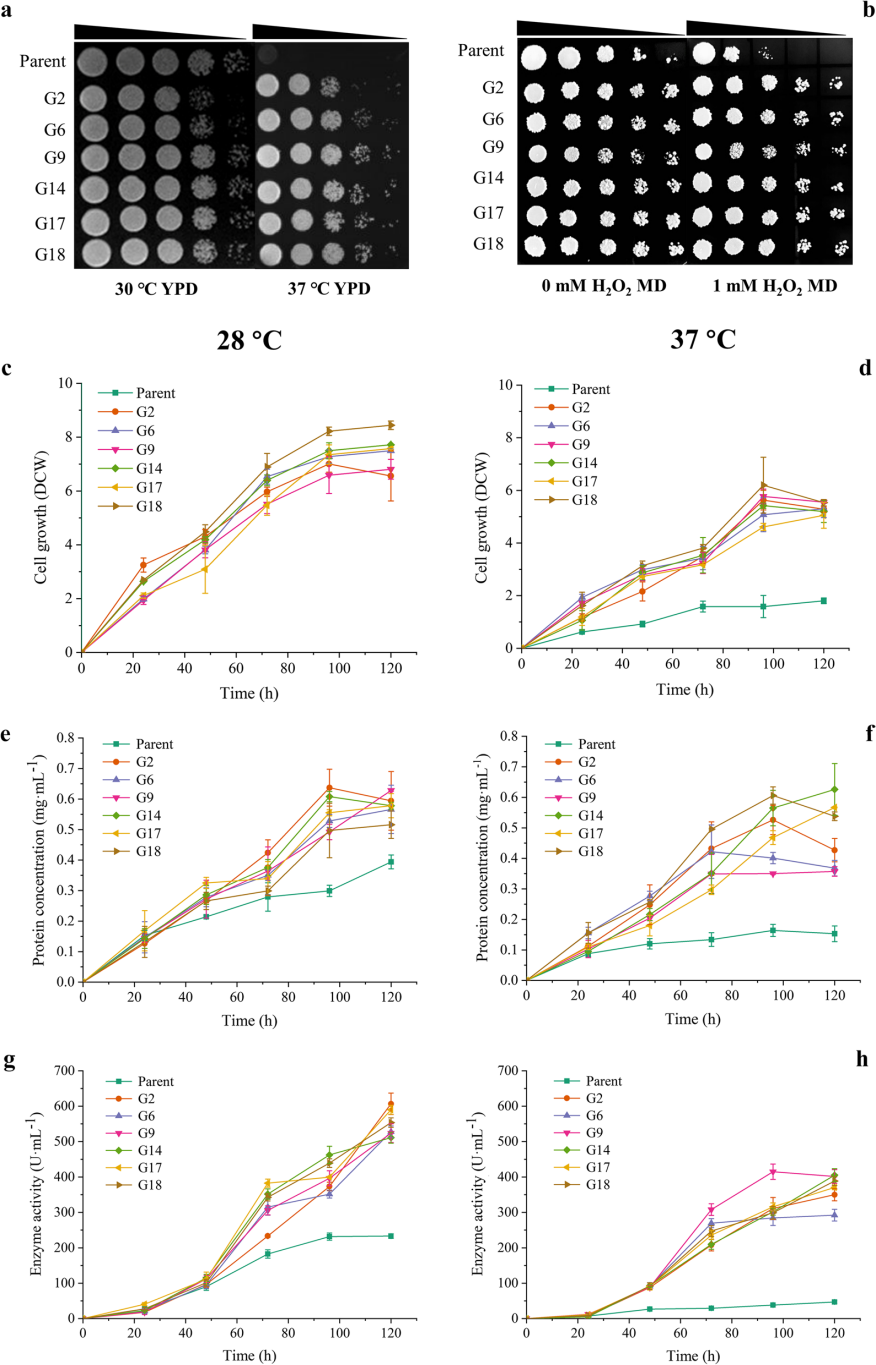
Cell growth test and fermentation with the parent and mutant strains. **a** YPD plates were used to test the impacts of thermal stress (37 °C) on the growth of the parent and mutants. **b** MD, and MD-H_2_O_2_ plates were used to test the impacts of oxidative stress (1 mM H_2_O_2_) on the growth of the parent and mutants; The lipase fermentation ability of the parent and mutants were test. Cell growth (**c**, **d**), protein concentration (**e**, **f**), and lipase activity (**g**, **h**) were measured and compared. **c**, **e**, **g** The fermentation condition is 28 °C. The growth levels of the parent and mutants were similar, while the protein concentration and enzyme activity of the fermentation broth in G14 was higher than that of the parent. **d**, **f**, **h** The fermentation condition is 37 °C. The protein expression levels of thermotolerant mutants were kept under thermal stress, while the parent can barely grow and express the heterologous protein. The error bars show the standard error of the mean based on three biological replicates

Adaptive evolution was used to breed thermotolerant strains. Yeasts with 5–10 °C higher growth temperature than their parents are labelled as thermotolerant yeasts [28]. The OD_600_ of the evolved cells after adaptive evolution at 37 °C was 1.3-fold higher than that of the parent at 30 °C after over 180 generations of temperature gradient domestication lasting for over 500 days (Additional file 1: Table S1). Six mutants were isolated from the evolved cells by olive oil/ rhodamine B screening plate. The six isolates retained genetic stability after a 45-day passage. As shown in the spot assay picture (Fig. 1a), the isolates were more tolerant to the high temperature than the parent, which can barely grow at 37 °C. Based on their growth performance in presence of thermal stress, these six isolates of yeast were thermotolerant.

Spot assay on MD solid medium added with 1 mM H_2_O_2_ was performed to examine the oxidative stress tolerance of the parent and thermotolerant mutants (Fig. 1b). The parent displayed a weak resistance to H_2_O_2_ damage compared with the thermotolerant mutants. The strong OSR in the mutants may prevent them from being damaged by oxidative stress and affected their cell growth.

Usually, thermotolerant yeast showed higher heterologous protein expression efficiency than its wild type [29]. To further explore thermotolerance and the lipase expression level of these thermotolerant isolates, batch-flask-fermentation was performed. As shown in Fig. 1c–h, following fermentation at non-stress and stress conditions (28 and 37 °C), dry cell weight, extracellular protein concentration, and lipase activity of these isolates and the parent were measured. At 28 °C, the cell growth of the thermotolerant strains was very similar to the parent (Fig. 1c). From the cell growth curve at 37 °C, we found that the thermotolerant strains showed better adaptability than the parent, which exhibited about a 3-fold higher dry cell weight (Fig. 1d). Interestingly, these isolates exhibited better lipase production ability than the parent at both non-stress and stress conditions (Fig. 1e, f). The lipase activities in the fermentation supernatant of the thermotolerant strains at 28 °C were over 2.5-fold than that of the parent (Fig. 1g). However, under the stress condition, the lipase expression ability of the parent was severely repressed (Fig. 1h). The results revealed that the heterologous protein expression ability of the thermotolerant isolates was kept under stress condition and even much better than that of the parent at non-stress fermentation condition. We chose G14 for further examination. In order to exclude the impact of lipase on the heat resistance of G14, we knocked out the gene encoding lipase and confirmed that the thermotolerance of G14-without lipase gene was still maintained (

Additional file 1: Fig. S1).

### ROS level, lipid peroxidation, and cell damage validation

**Fig. 2:**
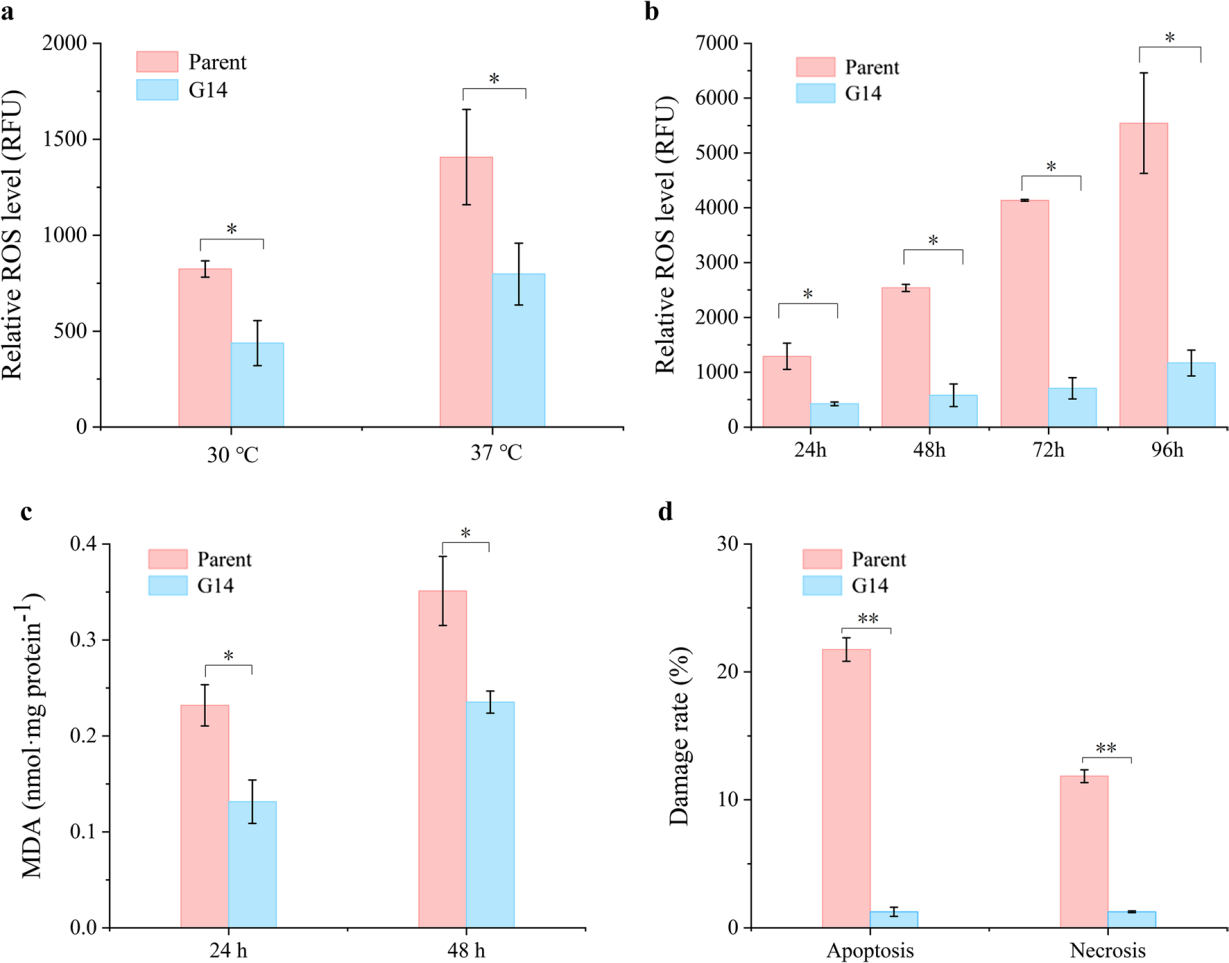
Cellular ROS level, MDA level, and cell damage of the parent and G14. **a** The relative ROS level of the parent and G14 grown at 30 °C for 16 h. The samples were collected at the logarithmic growth phase and treated with DCFH-DA at 30 °C (non-stress treatment condition) and 37 °C (stress treatment condition). Take the fluorescence intensity value of G14 treated at 30 °C as a reference. **b** ROS level of the parent and G14 during chronological aging at 37 °C. The fluorescence intensity value of G14 at 37 °C for 24 h was set as a reference. **c** Lipid peroxidation analysis of the parent and G14 by measuring MDA concentration under stress treatment condition. **d** The apoptosis and necrosis rate of cells under stress treatment condition. The early apoptotic cells were stained by Annexin V-FITC, while the necrotic cells were stained by Annexin V-FITC and PI simultaneously. Therefore, the percentage of apoptotic and necrotic cells after thermal stress was measured using flow cytometry. *P-value < 0.05, **P-value < 0.01. The error bars show the standard error of the mean based on biological replicates

Intracellular ROS and lipid peroxidation were measured to validate the oxidative stress induced by high temperatures. The ROS levels of G14 and the parent were measured under non-stress (30 °C) and stress conditions (37 °C) (Fig. 2a, b). Data from fluorescence intensity implied the ROS level. Under non-stress condition and treatment at 30 °C for 1 h, the parent had a 1.9-fold higher ROS level than G14 (Fig. 2a). Under non-stress condition but treatment at 37 °C for 1 h, the ROS level of the parent was still 1.8-fold higher than that of G14 (Fig. 2a). The ROS levels during chronological aging at 37 °C from 24 to 96 h were measured to check the impact of thermal stress on ROS levels. The fluorescence intensity gradually increased over time in both samples. In particular, the increasing trend of the parent was quite drastic, and that of G14 was relatively slow (Fig. 2b). The results revealed that the high temperature caused redox imbalance in cells and the ROS buffering capacity in G14 was stronger than that of the parent. Under oxidative stress, malondialdehyde (MDA) was generated as the end-product of cellular lipid peroxidation. As shown in Fig. 2c, the lower ROS level in G14 caused lower lipid peroxidation (1.5-fold lower) compared with the parent.

Environmental stresses usually cause cell damage and even cell death. Usually, stress-tolerant strains undergo a low ratio of cell apoptosis (early apoptosis) and necrosis (late apoptosis) under stress conditions [13]. The early apoptotic cells can be stained by Annexin V-FITC, while the necrotic cells can be stained by Annexin V- FITC and PI simultaneously. Therefore, the percentage of apoptotic cells and necrotic cells after thermal stress can be measured using flow cytometry. As shown in Fig. 2d and Additional file 1: Fig. S2, under stress condition, the cell apoptosis and necrosis rate in G14 were notably lower than those of the parent, 12.4- and 9.5-fold, respectively. In other words, the cell apoptosis and necrosis rate of *K. phaffii* mutant G14 upon stress condition were remarkably reduced after adaptive evolution.

Cell wall integrity is one of the important traits of thermotolerant strains [30]. Congo Red as a cell wall perturbing agent can be used to test the cell wall integrity of yeast. As shown in Additional file 1: Fig. S3, the parent was more sensitive to Congo Red at 30 °C than G14, implying the cell wall integrity of the parent was weaker than G14. Therefore, the improvement of cell wall integrity in G14 may positively affect its stress tolerance.

### Transcriptome analysis of thermotolerant strain G14 and the parent

**Fig. 3:**
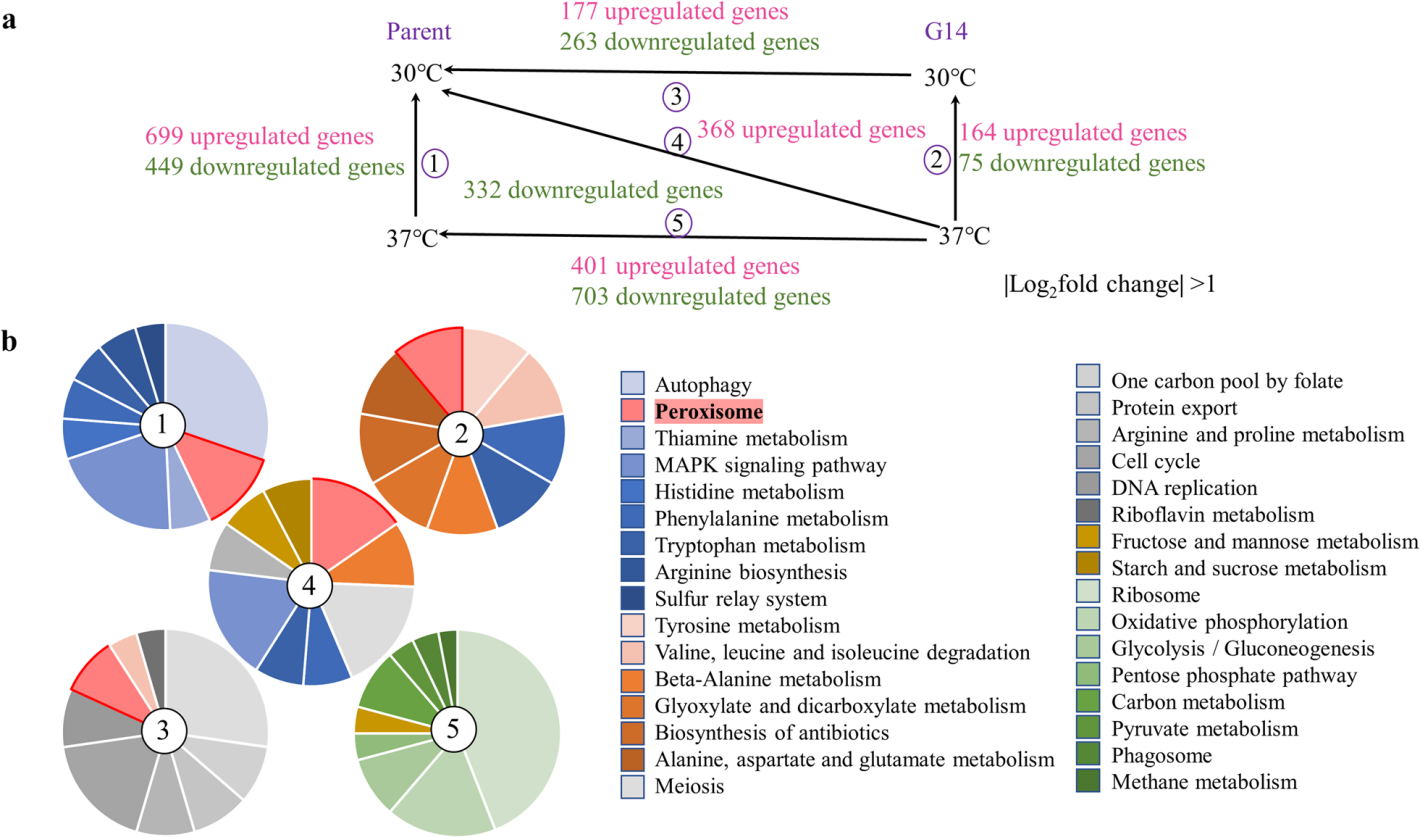
Transcriptome analysis of G14 and the parent. **a** The number of differently expressed genes in comparison groups. **b** The KEGG enrichment analysis of comparison groups. The top nine enriched upregulated pathways were depicted. The numbers 1–5 represent five comparison groups. 1: the parent under 37 °C versus the parent under 30 °C. 2: G14 under 37 °C versus G14 under 30 °C. 3: G14 under 30 °C versus the parent under 30 °C. 4: G14 under 37 °C versus the parent under 30 °C. 5: G14 under 37 °C versus the parent under 37 °C

Transcriptome analysis was conducted to further understand the thermotolerance mechanism of G14 from the molecular level. Three biological replicates were involved in RNA-sequencing (RNA-seq). The total raw data was uploaded in NCBI’s Gene Expression Omnibus (GEO) public archive database and the accession number is GSE157242. The overall quality of the data was assessed (Table S2). The correlation of samples was analysed by principal-component analysis (PCA) and analysis of similarities (ANOSIM). PCA analysis showed good repeatability of each biological replicate within each group. The results of PCA and ANOSIM showed that the data of RNA-seq in each group was significantly different (Additional file 1: Fig. S4). The results of the quality test indicated that the data could be employed for the following analysis. As shown in Fig. 3a, the differentially expressed genes between the parent and G14 were analysed at 30 and 37 °C. The samples were classified into five comparison groups for analysis. With the absolute value of log_2_ fold change > 1 as a classification criterion, the number of upregulated genes and downregulated genes of each comparison group are listed in Fig. 3a. Comparison group Parent_37 vs. Parent_30 with the largest number of differentially expressed genes represented the difference in gene expression levels of the parent at 37 °C versus at 30 °C. Given that the parent cannot adapt to thermal stress, its cellular changes were active. Comparison group G14_37 vs. G14_30 with the lowest number of differentially expressed genes was to reveal the changes in gene expression levels of G14 under 37 °C versus 30 °C. As a thermotolerant strain, G14 did not have to alter much to adapt to thermal stress. Comparison groups G14_30 vs. Parent_30 and G14_37 vs. Parent_37 was used to explore the differentially expressed genes between G14 and the parent under the same temperature. As shown in Fig. 3a, the number of downregulated genes was higher than that of upregulated genes in comparison G14_30 vs. Parent_30 and G14_37 vs. Parent_37. The reason might be the same as that for comparison group Parent 37 °C vs. Parent 30 °C. Comparison group G14_37 vs. Parent_30 was to reveal the differentially expressed genes of G14 at 37 °C versus those of the parent at 30 °C, showing the changes of the evolved strain under thermal stress versus the parent under non-stress condition.

### Peroxisome pathway plays an important role in the thermotolerance of G14

To explore the functional pathways in G14, upregulated genes of five comparison groups were analysed using Kyoto Encyclopedia of Genes and Genomes (KEGG) pathway enrichment analysis. The top nine enriched upregulated pathways are summarized in the pie charts (Fig. 3b). The results revealed that the peroxisome pathway was one of the most significantly upregulated pathways in comparison group Parent_37 vs. Parent_30, G14_37 vs. G14_30, G14_30 vs. Parent_30, and G14_37 vs. Parent_30. The phenylalanine metabolism, tryptophan metabolism, and mitogen-activated protein kinase (MAPK) pathway were also enriched by KEGG pathway enrichment analysis.

**Fig. 4:**
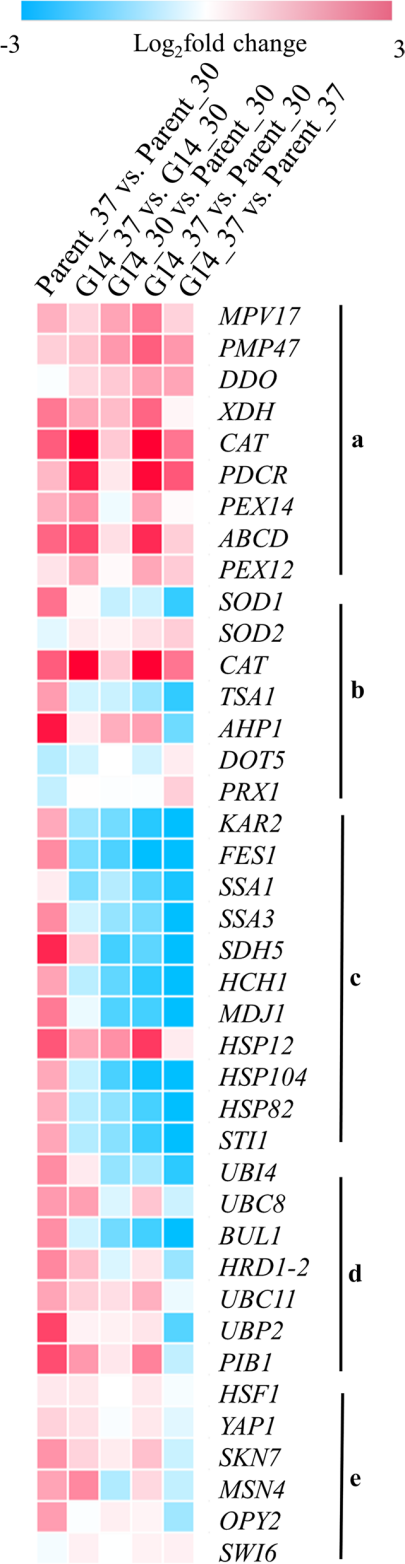
Transcriptional profile of related genes. **a** Peroxisomal protein encoding genes. **b** Genes related to antioxidant defence system. **c** HSPs and HSP related genes. **d** Ubiquitin and ubiquitin related genes. **e** Transcription factors

As the peroxisome pathway was upregulated in four comparison groups, the transcription profile of upregulated peroxisomal protein encoding genes were explored (Fig. 4a, Additional file 1: Table S3). The expression levels of key peroxisomal protein encoding genes measured by RT-qPCR (Additional file 1: Fig. S5) were consistent with the results of RNA-seq. Peroxisomal catalase encoded by *CAT* is an important enzyme that reduces cellular oxidative damage [31]. As shown in Fig. 4a, the transcription levels of *CAT* were significantly increased in all comparison groups. In comparison group G14_37 vs. Parent_37, the expression level of *CAT* was upregulated to 1.6 Log_2_foldchange. The catalase activity of the soluble protein extracts was examined according to the degradation of H_2_O_2_ [31]. The results of catalase activity were consistent with expression levels that the catalase activity of G14 at 30 °C (2.97 U/mg protein) and 37 °C (5.27 U/mg protein) was 3.9- and 2.5-fold higher than that of the parent (0.76 U/mg protein and 2.14 U/mg protein), respectively. And, in peroxisome biogenesis process, the upregulated gene *MPV17* is involved in ROS metabolism, thereby explaining the low ROS level and resistance to H_2_O_2_ stress in G14.

**Fig. 5:**
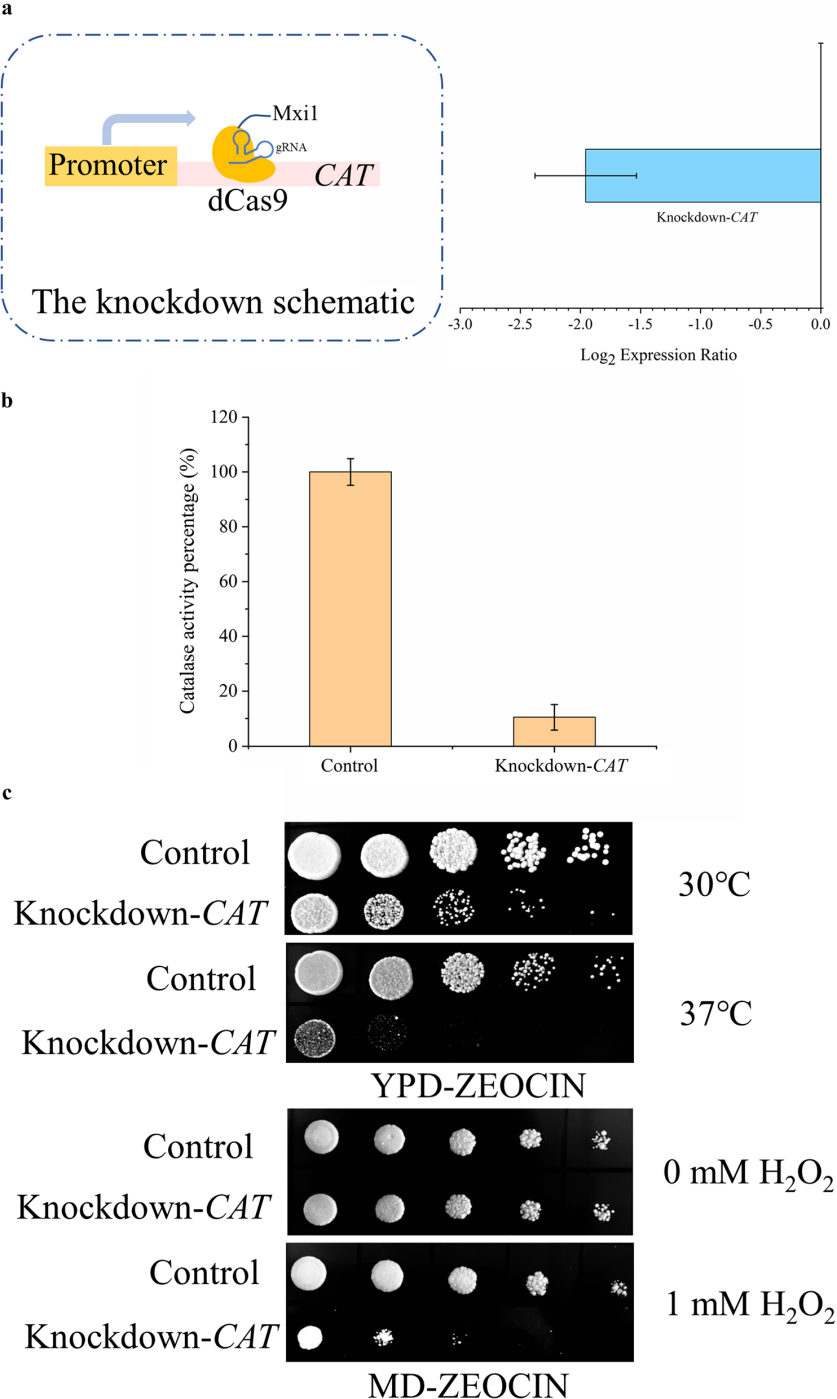
The gene knockdown of ***CAT***. **a** The schematic of gene knockdown was showed in the left side, the dCas9 with Mxi1 repressor was targeted to the N-terminus of *CAT* to repress the function of *CAT*. As showed in the right side, the efficiency of gene knockdown experiment was detected by RT-qPCR. **b** The catalase activity was measured and compared to assess the repression efficiency of dCas9 system. **c** Spot assay to test the impact caused by gene knockdown of *CAT* on cell growth of G14 at different temperature and with/without H_2_O_2_

A gene knockdown experiment was performed to further explore the contribution of catalase on the oxidative and thermal stress cross-tolerance of G14. An episomal plasmid with a guide RNA was constructed to target dCas9 protein to the *N*-terminal region of *CAT* in G14 (Fig. 5a). Simultaneously, a control was transformed with an episomal plasmid with no guide RNA. As shown in Fig. 5a, the expression level of *CAT* was significantly repressed after knockdown. The catalase activity was reduced to 10.5 % of the control (Fig. 5b). Interestingly, the thermal and oxidative stress cross-tolerance of G14 was tremendously repressed that the *CAT* knockdown strain can barely grow under 37 °C and H_2_O_2_ medium (Fig. 5c). To cultivate cells with episomal plasmids without adding resistance reagent, the episomal plasmids will be lost soon. Therefore, we promoted the loss of episomal plasmid by not adding resistance to confirm whether the loss of plasmid restores thermotolerance of G14. We identified that the thermal and oxidative stress cross-tolerance of G14 was fully restored after plasmid loss, as expected (Additional file 1: Fig. S6). The *CAT*-knockdown plasmid was introduced into the parent (Additional file 1: Fig. S7), cells grew normally without stress treatment, but still cannot grow under stress conditions as before.

**Fig. 6:**
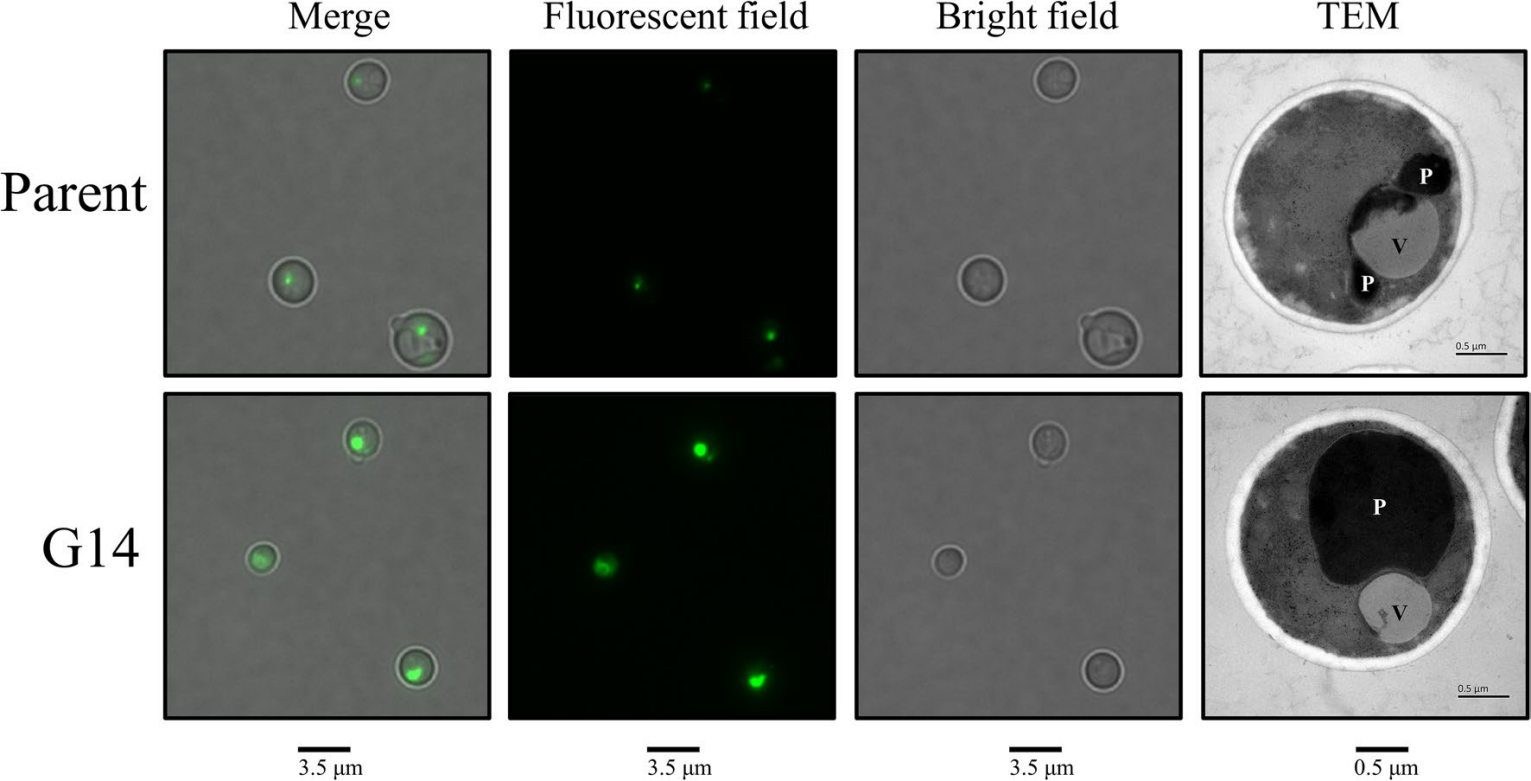
Fluorescence microscopy analysis and transmission electron microscopy (TEM) analysis. For fluorescence microscopy analysis, the peroxisomal membranes are marked by *PMP47*-*GFP* fusion protein. Hence, the volume of fluorescence spot indicates the volume of peroxisome in each sample. P, peroxisomes; V, vacuole. Scale bars, 3.5 μm (Merge, Fluorescent field, Bright field), 0.5 μm (TEM)

The peroxisomal membrane gene *PMP47* was significantly upregulated in all comparison groups. We tagged the *PMP47* with a gene encoding green fluorescent protein (GFP) to visualize the change of peroxisome in G14 compared to the parent. As shown in Fig. 6, the larger fluorescence spots of PMP47-GFP fusion protein in G14 indicated that there may be larger peroxisomes in G14 than that in the parent. The fluorescence intensity of GFP in G14 was 4-fold higher than that in the parent. Additionally, this phenomenon was consistent with the TEM analysis. We identified organelles according to Aksam et al. [32]. In theory, the larger the peroxisome, the more peroxisomal enzymes are contained [33]. More peroxisomal enzymes were hypothesized to be embedded in the peroxisomes of G14 than the parent. Thus, we inferred that maybe the larger peroxisomes leading to stronger peroxisomal ROS buffering capacity renders the oxidative and thermal stress cross-tolerance in G14. Interestingly, we also found the cell volume of G14 was moderately smaller than the parent. Other researchers have found that the composition change in membrane [34] or cell wall [35] is generally associated with the improvement of stress tolerance in industrial strains. Therefore, we inferred that the cell membrane composition and cell wall composition were changed in the G14. The difference of cell membrane and cell wall between the parent and G14 remains to be further studied in the future.

### Changes in expression levels of genes related to antioxidant defence system, HSPs, ubiquitin, and transcription factors

To our knowledge, function factors, such as antioxidants, HSPs, ubiquitin, and transcription factors are responsible for environmental stress tolerance in yeast. Therefore, the expression levels of key genes that possibly contribute to thermotolerance were analysed [5] (Fig. 4; Additional file 1: Table S3). Some of the genes related to antioxidants were upregulated in all comparison groups (Fig. 4b). In comparison group G14_37 vs. G14_30 and G14_37 vs. Parent_30, most of the listed HSP-related genes (Fig. 4c) did not undergo transcriptional upregulation but were significantly downregulated. The expression levels of some ubiquitin-related genes (Fig. 4d) were only slightly upregulated. Hence, the antioxidant defence system and some ubiquitin-related genes, but not the HSPs, were responsible for the thermotolerance and oxidative stress tolerance of G14. Although oxidative stress is considered to be secondary stress under thermal conditions [36], in fact, according to our research, we believe that once high temperature is encountered, cells will be immediately impacted by oxidative stress. The antioxidant defence system is controlled by OSR, and HSPs are mediated by HSR. In the parent, according to the result of comparison group Parent_37 vs. Parent_30, both these two resistance systems (OSR and HSR) were induced, while in G14 only OSR was induced. Based on the current results, under thermal stress, OSR may be induced prior to HSR, when it fails to overcome, the cells start the protective program of HSR.

In comparison group Parent_37 vs. Parent_30, the parent at 37 °C versus at 30 °C, the most significantly upregulated pathway was autophagy (Fig. 3b). The finding was consistent with the severe cell apoptosis and death in the parent under thermal stress. However, the peroxisome pathway was also significantly upregulated in comparison group Parent_37 vs. Parent_30. The parent had higher cellular ROS levels but considerably lower catalase activity than G14. These results indicated that the ROS buffering capacity in the parent was not strong enough. With the failed OSR in the parent, the HSR started. As shown in Fig. 4c, most genes related to HSPs were significantly upregulated in comparison group Parent_37 vs. Parent_30. In addition, all listed ubiquitin relative genes were considerately upregulated (Fig. 4d), proving that thermal stress leads to the aggregation of toxic proteins that need to be marked by the ubiquitin and then degraded. Transcription factors who are responsible for environmental stress tolerance were upregulated in comparison group Parent_37 vs. Parent_30, G14_37 vs. G14_30, and G14_37 vs. Parent_30 (Fig. 4e), showing normal response of yeast under thermal stress. However, owing to the contribution of catalase, this trend in comparison group G14_37 vs. Parent_37 was different.

## Discussion

The role of redox balance in oxidative stress has been extensively studied. Thermal stress also induces oxidative stress in cells, so redox balance contributes to thermotolerance as well. HSR and OSR play crucial roles under thermal stress, but their coordinated regulation of thermotolerance mechanism is still unclear. Thermotolerance is one of the desirable features for yeasts during fermentation production. Our results indicated that the OSR in G14 induced strong peroxisomal ROS buffering, which helps promote its thermal and oxidative stress cross-tolerance. Nevertheless, the upregulation of antioxidant defence systems in the parent failed to reduce thermal stress-induced cellular ROS levels in time. The parent had to start HSR, leading to the upregulation of HSP-related genes. However, the cells cannot recover, thereby inducing the autophagy pathway and resulting in severe cell death. Therefore, we boldly speculate that OSR and HSR in yeast are activated in order upon thermal stress: OSR is activated first, and if it fails, HSR is activated. This inference still needs more research to prove.

OSR is responsible for preventing cell damages from loss of physiologically proper redox balance. To our knowledge, environmental stresses trigger ROS generation, which causes redox imbalance, damage cellular components, and reduce cell viability [7, 37, 38]. Cellular ROS accumulation aggregates denatured protein [39] and consequently disturbs the normal protein expression [40]. The antioxidant defence system scavenges the ROS generated in yeast cells and requires the presence of antioxidants such as superoxide dismutase, catalase, thioredoxins, peroxiredoxin, and glutathione [16, 41–44]. Peroxisomes are responsible for cellular ROS generation and detoxification [33, 45]. Catalase, known as a ROS scavenger, is mostly localized in peroxisomes [46]. In xylose alcoholic fermentation, the active fission of peroxisomes and an increase of peroxisomes size enhances the efficient ROS detoxification [33]. Overexpression of peroxisomal biogenesis gene *PEX34* improves xylose alcoholic fermentation efficiency [33]. Consistent with these results, the peroxisomes size in G14 was larger than that in the parent (Fig. 6), implying a higher level of catalase. *K. phaffii* can utilize methanol as the sole carbon resource to produce heterologous proteins. The function of peroxisomes and methanol utilization pathway is responsible for methanol catabolism [47, 48]. Therefore, the peroxisome pathway might be an important target to improve stress tolerance and protein expression of *K. phaffii*.

In yeast, HSPs maintain protein structure. The synthesis of HSPs is controlled by HSR with the heat shock transcription factor 1 (*HSF1*) as the primary regulator [12]. The thermotolerant mutant and the parent had slightly induced *HSF1* under thermal stress, whereas the expression levels of most HSP-related genes were considerably different (Fig. 4c). Although many HSP-related genes were highly upregulated, the parent still failed to achieve thermotolerance. It may because of the protection from HSPs is only short-acting [12] that once the duration of thermal stress is enhanced, the cells ultimately undergo cell death. Although the overexpression of HSP has been applied to improve the thermotolerance [49, 50], the effects are not ideal. We assumed that preventing protein misfolding by OSR is more important than the repair by HSR after damage. This study provides evidence that the OSR in the programmed thermotolerance mechanism is to reduce oxidative stress and prevent the formation of misfolded proteins and thus is the key to improving the thermotolerance of yeast.

Laboratory evolution is one of the operative techniques to associate genotypes with phenotypes. After genome sequencing, we found many gene mutations in G14 relative to the parent. The genome sequencing data will be correlated to RNA-seq data to analyse the contribution of specific gene mutations to the thermotolerance and oxidative stress tolerance of *K. phaffii* in the future and facilitate the rational engineering of robust strains. Based on RNA-seq results, overexpression or down-regulation of the identified gene targets will be carried out in the parent to evaluate their contributions to thermotolerance.

## Conclusions

This study revealed the possible contribution of peroxisomes in the thermotolerance of yeast. A stronger OSR with high expression of peroxisomal catalase may protect yeast from thermal and oxidative stress. Our findings provide new insight into the thermotolerance mechanism of yeast and highlight the potential method for constructing thermotolerant yeasts by enhancing its ability to remove cellular ROS and maintain redox balance.

## Methods

### Strains and breeding method

The *K. phaffii* used as the parent is a recombinant GS115 constructed previously in our laboratory, expressing a recombinant lipase [51]. Usually, for cell growth, GS115 is cultured at 30 °C, for heterologous protein expression, the temperature is 28 °C. The breeding method was modified according to Satomura et al. [52]. Cells were treated with atmospheric and room temperature plasmas (ARTP) [53] before adaptive evolution to acquire potential mutations. The lethal rate was controlled at 90%. An aliquot of pre-cultured ARTP treated cells was inoculated into a 250 mL shake-flask with 100 mL of fresh YPD medium at 32 °C. For the control, the parent was grown at 30 °C. The initial OD_600_ of samples was 0.1. OD_600_ for both was measured after 3 days. The OD_600_ ratio between the cells at the high acclimation temperatures (32-38 °C) and the parent at non-stressed condition (30 °C) was analysed to evaluate whether the cells adapt to high acclimation temperature. This adaption process was repeated several rounds until the evolved cells adapted to 32 °C, that is, the OD_600_ ratio was stably over 1 for three generations. The cells were stepwise adapted to 34 °C, 36 °C, and 38 °C. Olive oil/rhodamine B screening plate (1% olive oil and Polyvinyl Alcohol emulsion, 100 mM potassium phosphate (pH 6.0), 1.34% yeast nitrogen base without amino acids, 4 × 10^− 5^% biotin, 1 × 10^− 3^% rhodamine B, 0.5 % methanol) was used to isolate thermotolerant and lipase productive strains from the evolved cells under 37 °C. Briefly, the series of tenfold diluted culture was diluted and plated on olive oil/rhodamine B screening plate. The lipase secreting from the colony catalyses the olive oil into fatty acid, which reacts with rhodamine B to form a red visible circle around the colony. The volumes of red visible circles indicate the lipase activity of single colonies. Hence, the colonies with large red visible circle were isolated for further exploration. After that, the thermotolerant isolates were cultured at non-stress condition (30 °C) for a 45-day passage and then switched to be cultured at stress condition (37 °C) to test their genetic stability.

### Spot assay

For spot assay, yeast cells were cultivated in 5 mL of YPD medium at 30 °C to mid-logarithmic phase. Cell pellets were harvested through centrifugation and washed with sterile saline. The OD_600_ of cell suspension was adjusted to 1.0. Ten-fold serial dilutions of cell suspension were prepared for spot assay. Heat sensitivity, oxidative stress sensitivity, and cell wall integrity were studied by spotting equal amounts of cells onto solid YPD plate, MD (1.34% yeast nitrogen base, 4 × 10^− 5^% biotin, 2 % glucose) solid medium supplemented with 1 mM H_2_O_2_, and YPD solid medium supplemented with 100 µg/mL Congo Red. Photographs were taken after 3 days of incubation.

### Expression of the recombinant lipase

The lipase expression by the parent and the isolates were performed according to Sha et al. [51]. Briefly, a single colony of each sample was picked and inoculated in BMGY/BMMY fermentation medium at 28 and 37 °C for batch-flask-fermentation. The expression of the lipase was induced by methanol every 24 h. And, 1 mL of cell culture was collected for further determination every 24 h. The culture supernatants were collected for protein concentration and lipase activity determination [51]. The protein concentration was measured by Bradford method [54]. Briefly, the lipase activity was measured on emulsified p-nitrophenyl palmitate (pNPP). The variation of the absorbance at 410 nm of the assay against a blank was monitored for 2 min using a microplate reader (BioTek, USA). One enzyme unit was defined as the amount of enzyme releasing 1 µmol of p-nitrophenol per minute under the assay conditions.

### ROS level, MDA concentration, and cell damage determination

Intracellular ROS was measured using 2,7-dichlorodihydrofluoroscein diacetate (DCFH-DA) [55]. The cells were harvested through centrifugation, resuspended in PBS, and treated in 10 µM DCFH-DA dissolved in DMSO for 1 h at 30 °C or 37 °C. After centrifugation, the cell pellets were resuspended in PBS and crushed by glass bead disruption. The supernatant was collected, and DCF fluorescence intensity was measured at an excitation wavelength of 480 nm and an emission wavelength of 525 nm. MDA assay kit (Solarbio, China) was used to measure MDA [7] concentration. A total of 5 × 10^7^ cells were harvested through centrifugation, and the cell pellets were resuspended in extraction buffer and crushed by glass bead disruption. The supernatant was collected for further determination as instructed in the user manual. The protein concentrations of both supernatants were measured by Bradford method [54]. DCF fluorescence intensity was normalized to the protein level of the supernatant. Three biological replicates of each sample were adopted to ensure data reliability.

A total of 2.5 × 10^7^ cells were harvested for the assessment of apoptosis and necrosis [13] by Annexin V-FITC Apoptosis Detection Kit (Beyotime, Shanghai, China). The cells were analysed by Flow cytometry (BD, USA) with an excitation wavelength of 488 nm and an emission wavelength of 515–535 nm. Before staining, the cells were treated with enzyme solution (1 % cellulase, 0.5 % snail enzyme, 0.25 % lysozyme) for 1.5-2 h. The staining process was performed following the manufacturer’s recommendations. After staining, the samples must be detected within 1 h. The raw data of flow cytometry were analysed by FlowJo software (FlowJo, USA). Statistical analysis was performed with Microsoft Office Excel 2019 (Microsoft).

### RNA-sequencing

Samples for RNA-seq were collected from cell cultures in YPD medium under 30 or 37 °C at the logarithmic phase. Three biological replicates of each sample were adopted to ensure data reliability. The cDNA library construction and sequencing service were provided by Novogene, Beijing, China (http://www.novogene.cn/). Sequencing libraries were generated using NEBNext^®^ Ultra™ RNA. Library Prep Kit for Illumina^®^ (NEB, USA) following the manufacturer’s recommendations. All samples were sequenced on an Illumina NovaSeq 6000 platform and 150 bp paired-end reads were generated. For the reads mapping, the reference genome, and gene model annotation files of *K. phaffii* were downloaded from PICHIAGENOME database (http://pichiagenome-ext.boku.ac.at:8080/apex/f?p=100:1:12609695393049::NO::::YES). Raw data (raw reads) of fastq format were firstly processed through in-house Perl scripts. In this step, clean data (clean reads) were obtained by removing reads containing adapter, reads containing ploy-N, and reads with low quality from raw data. At the same time, Q20, Q30 of the clean data were calculated. All the downstream analysis was based on the clean data with high quality. The resulting *P*-values were adjusted using the Benjamini and Hochberg’s approach for controlling the false discovery rate. Genes with an adjusted *P*-value < 0.05 found by DESeq2 were assigned as differentially expressed. In order to evaluate the differences between groups and the repetition of samples within groups, PCA was performed. ANOSIM was carried out in R with the vegan package to further test group differences based on Bray-Curtis distance matrices [56]. PCA and KEGG pathway enrichment analysis were implemented by the Novogene cloud platform (https://magic.novogene.com).

### RNA isolation and RT-qPCR

Transcription level of genes were examined by RT-qPCR. Cells of logarithmic growth phase were collected. Cell pellets were immediately frozen in liquid nitrogen. Total RNA was extracted using Yeast RNAiso Kit (Takara, China). The PrimeScript™ RT reagent Kit with gDNA Eraser (Perfect Real Time) from Takara was used to synthesize cDNA. The *actin* gene served as a reference gene. Amplification primers of genes were listed in Additional file 1: Table S4. Amplification was achieved using 2*SG Fast qPCR Master Mix (High Rox) (Sangon Biotech, China) in the ABI StepOne Plus Real-Time PCR System (ABI, Germany). All experiments were independently performed in triplicate. Relative expression level of gene was analysed by the 2^−[delta][delta]Ct^ method [57].

### Catalase activity assay

Catalase activity was assayed by measuring the degradation of H_2_O_2_ at 240 nm using the extinction coefficient for H_2_O_2_ of 39.4 M^− 1^ cm ^− 1^ [58]. One unit of catalase activity catalyzed the disappearance of 1 µmol of H_2_O_2_ per min. Briefly, a total of 1 × 10^8^ cells were harvested and suspended in PBS and then lysed by glass bead disruption. After centrifugation, the protein concentrations of supernatants were measured by Bradford method. A 200 µL of the extract was added to the H_2_O_2_ reaction system. The absorbance at 240 nm was recorded at 0 and 1 min.

### Plasmid construction

For the construction of *PMP47-GFP* plasmid, the sequence of *PMP47* was cloned from genome of *K. phaffii*. A GFP sequence was linked at the C-terminal of *PMP47* with a linker, 5’-GGTGGTGGTGGTTCTGGTGGTGGTGGTTCT-3’. For the construction of episomal knockdown plasmid, a dCas9 (D10A; H840A) linked with a Mxi1 repressor and gRNA scaffold cassette were acquired by gene synthesis (Sangon Biotech, Shanghai) according to Weninger et al. [59]. The schematic of dCas9 system was showed in Fig. 5a. A guide RNA (gRNA), 5’-TTCCACCAAGTTTTACACAG-3’, was constructed into the knockdown plasmid to target the N-terminal region of *CAT*. For the construction of episomal lipase gene knockout plasmid, Cas9 and gRNA (5’-ATATTAACAAGAGCGTTCAA-3’) were also acquired by gene synthesis according to Weninger et al. [59]. The knockout of lipase gene was confirmed by PCR and sequencing. The inactivation of lipase gene was further confirmed by plating gene knockout strains on olive oil/rhodamine B screening solid medium. A control was constructed with no guide RNA. These plasmids carried zeocin resistance cassette. The Zeocin™ selection antibiotic was purchases from Thermo Fisher (Thermo Fisher, USA). The rescue of the episomal plasmid was carried out by culturing cells for a few rounds in medium plate without zeocin selection antibiotic until cells could not grow on zeocin selection plate.

### Fluorescence microscopy and transmission electron microscopy

Fluorescence microscopy was performed on a Leica DMi8 fluorescence microscope (Leica, Germany). GFP signal was visualized with a 460–500 nm bandpass excitation filter, a 505-nm dichromatic mirror, and a 512–542 nm bandpass emission filter. Over 200 cells of each sample were randomly chosen for observation. For TEM, cells were harvested at the logarithmic growth phase. The TEM sample preparation method was as follows: cells were pre-fixed with 5 % glutaraldehyde, and then rinsed with 0.1 M phosphate buffer; next, cells were fixed with 1 % osmic acid and rinsed with 0.1 M phosphate buffer; gradient dehydration of cells with ethanol was performed before soaked and embedded with epoxy resin. Finally, the ultra-thin sections of prepared cell samples were placed under a transmission electron microscope (HITACHI H-7650, Hitachi, Japan) for observation. Over 100 cells of each sample were randomly chosen for observation.

## Supplementary Information

**Additional file 1**: **Table S1** Number of generations for each temperature gradient. Table S2 Overall quality of the generated sequencing data for each group. Table S3 The information of genes in Fig. 4. Table S4 List of genes and primers for RT-qPCR used in this study. **Fig. S1**. Spot assay to show the impact of lipase gene in the thermotolerance of G14. **Fig. S2**. The Annexin V-FITC/PI staining was analyzed by flow cytometry for cell apoptosis and necrosis. **Fig. S3**. The test of cell wall integrity by Congo Red resistance test. **Fig. S4**. The correlation of samples by principal-component analysis (PCA) and analysis of similarities (ANOSIM). **Fig. S5**. Expression level of peroxisomal protein encoding genes in five comparison groups. **Fig. S6**. Spot assay to show the stress tolerance of G14 was recovered after losing *CAT* gene knockdown episomal plasmid. **Fig. S7**. Spot assay to test the impact caused by gene knockdown of *CAT* on cell growth of the parent at different temperature and with/without H_2_O_2_.

## Data Availability

Not applicable.
