# Supplementary material for: Augmented peroxisomal ROS buffering capacity renders oxidative and thermal stress cross-tolerance in yeast
Source: Microb Cell Fact. 2021 Jul 12;20:131. doi: 10.1186/s12934-021-01623-1 (PMC8273976; doi:10.1186/s12934-021-01623-1)
Supplement: Supplementary file 1 — Additional file 1: Table S1 Number of generations for each temperature gradient. Table S2 Overall quality of the generated sequencing data for each group. Table S3 The information of genes in Fig. 4. Table S4 List of genes and primers for RT-qPCR used in this study. Fig. S1. Spot assay to show the impact of lipase gene in the thermotolerance of G14. Fig. S2. The Annexin V-FITC/PI staining was analyzed by flow cytometry for cell apoptosis and necrosis. Fig. S3. The test of cell wall integrity by Congo Red resistance test. Fig. S4. The correlation of samples by principal-component analysis (PCA) and analysis of similarities (ANOSIM). Fig. S5. Expression level of peroxisomal protein encoding genes in five comparison groups. Fig. S6. Spot assay to show the stress tolerance of G14 was recovered after losing CAT gene knockdown episomal plasmid. Fig. S7. Spot assay to test the impact caused by gene knockdown of CAT on cell growth of the parent at different temperature and with/without H2O2. [file 12934_2021_1623_MOESM1_ESM.docx]

**Additional File 1**

**Augmented peroxisomal ROS buffering capacity renders****oxidative and thermal stress cross-tolerance in yeast**

Nai-Xin Lin, Rui-Zhen He, Yan Xu, and Xiao-Wei Yu^^[[1]](#footnote-1)^*^

*Key Laboratory of Industrial Biotechnology, Ministry of Education, School of Biotechnology, Jiangnan University, Wuxi 214122, PR China*

TABLES

**Table S1 Number of generations for each temperature gradient**

| Temperature | OD_600_ Ratio | Generations |
| --- | --- | --- |
| From 30 °C to 32 °C | 1.12 | 6 |
| From 32 °C to 34 °C | 1.17 | 16 |
| From 34 °C to 36 °C | 1.26 | 38 |
| From 36 °C to 38 °C | 1.34 | 126 |

OD_600_ Ratio: the OD_600_ ratio between the cells at the high acclimation temperatures (32 °C-38 °C) and the parent at non-stressed condition (30 °C). When the ratio was stably over 1 for three generations, we deemed that the cells has adapted to the temperature, and then switch next acclimation temperature.

Generations: the number of generations required for the cells to adapt to the temperature.

**Table S2 Overall quality of the generated sequencing data for each group**

| Sample name | Raw reads | Clean reads | Total map | Clean bases | Error rate (%) | Q20 (%) | Q30 (%) |
| --- | --- | --- | --- | --- | --- | --- | --- |
| Parent_30_1 | 33993002 | 33015202 | 31202366(94.51%) | 4.95G | 0.03 | 97.77 | 93.69 |
| Parent_30_2 | 31070996 | 30038964 | 28619276(95.27%) | 4.51G | 0.03 | 97.48 | 93.09 |
| Parent_30_3 | 39582658 | 38402992 | 36753707(95.71%) | 5.76G | 0.03 | 97.75 | 93.65 |
| G14_30_1 | 31014942 | 30066370 | 28888350(96.08%) | 4.51G | 0.03 | 97.71 | 93.57 |
| G14_30_2 | 50127200 | 48920978 | 47199229(96.48%) | 7.34G | 0.03 | 97.74 | 93.6 |
| G14_30_3 | 46515544 | 45331768 | 43870140(96.78%) | 6.8G | 0.03 | 97.91 | 93.98 |
| Parent_37_1 | 35765524 | 35236674 | 32978488(93.59%) | 5.29G | 0.03 | 97.12 | 92.26 |
| Parent_37_2 | 35613524 | 34520228 | 32129036(93.07%) | 5.18G | 0.03 | 97.38 | 92.88 |
| Parent_37_3 | 51576390 | 50131188 | 47048997(93.85%) | 7.52G | 0.03 | 97.54 | 93.22 |
| G14_37_1 | 35618554 | 34615440 | 33199922(95.91%) | 5.19G | 0.03 | 97.82 | 93.82 |
| G14_37_2 | 41040000 | 40594014 | 39051765(96.2%) | 6.09G | 0.03 | 97.2 | 92.09 |
| G14_37_3 | 35579878 | 34630986 | 33344584(96.29%) | 5.19G | 0.03 | 97.51 | 93.12 |

Error rate: sequencing error rate. Q20: the bases amount ratio of error rate ≤ 1%. Q30: the bases amount ratio of error rate ≤ 0.1%.

**Table S3** **The information of genes in Fig. 4**

| Gene name | Gene ID | Gene description |
| --- | --- | --- |
| **Peroxisomal protein encoding genes** | | |
| *MPV17* | 8198677 | Protein required for ethanol metabolism |
| *PMP47* | 8199380 | Peroxisome membrane protein 47 |
| *DDO* | 8199538 | D-aspartate oxidase |
| *XDH* | 8199216 | Putativexanthine dehydrogenase |
| *CAT* | 8198267 | Catalase |
| *PDCR* | 8199722 | Peroxisomal C4-dienoyl-CoA reductase, auxiliary enzyme of fatty acid beta-oxidation |
| *PEX14* | 8200572 | Peroxisomal membrane peroxin |
| *PXA* | 8198676 | Subunit of a heterodimeric peroxisomal ATP-binding cassette transporter |
| *PEX12* | 8200652 | RING-finger peroxisomal membrane peroxin |
| **Genes related to antioxidant defense system** | | |
| *SOD1* | 8200564 | Cytosolic superoxide dismutase |
| *SOD2* | 8197288 | Mitochondrial superoxide dismutase |
| *CAT* | 8198267 | Peroxisomal catalase |
| *TSA1* | 8198279 | Thioredoxin peroxidase |
| *AHP1* | 8197158 | Thiol peroxidase |
| *DOT5* | 8196659 | Nuclear thiol peroxidase |
| *PRX1* | 8200260 | Mitochondrial peroxiredoxin |
| **HSPs and HSP related genes** | | |
| *KAR2* | 8198455 | A chaperone to mediate protein folding |
| *FES1* | 8198161 | Hsp70 (Ssa1p) nucleotide exchange factor |
| *SSA1* | 8200820 | Member of HSP70 family |
| *SSA3* | 8199979 | ATPase involved in protein folding and the response to stress |
| *SDH5* | 8199011 | Essential Hsp90p co-chaperone |
| *HCH1* | 8200190 | Co-chaperone that binds to Hsp82p and activates its ATPase activity |
| *MDJ1* | 8198223 | Co-chaperone that stimulates the ATPase activity of the Hsp70 protein Ssc1p |
| *HSP12* | 8197412 | Plasma membrane protein involved in maintaining membrane organization in stress conditions |
| *HSP104* | 8197345 | Heat shock protein that cooperates with Ydj1p (Hsp40) and Ssa1p (Hsp70) |
| *HSP82* | 8201336 | Heat shock protein Hsp90 |
| *STI1* | 8198440 | Hsp90 co-chaperone |
| **Ubiquitin and ubiquitin related genes** | | |
| *UBI4* | 8200655 | Ubiquitin |
| *UBC8* | 8199657 | Ubiquitin-conjugating enzyme that negatively regulates gluconeogenesis |
| *BUL1* | 8198980 | Ubiquitin-binding component of the Rsp5p E3-ubiquitin ligase complex |
| *HRD1-2* | 8200726 | Ubiquitin-protein ligase |
| *UBC11* | 8200408 | Ubiquitin-conjugating |
| *UBP2* | 8199397 | Ubiquitin-specific protease that removes ubiquitin from ubiquitinated proteins |
| *PIB1* | 8196791 | RING-type ubiquitin ligase of the endosomal and vacuolar membranes |
| **Transcription factors** | | |
| *HSF1* | 8198145 | Heat shock transcription factor |
| *YAP1* | 8200866 | Basic leucine zipper (bZIP) transcription factor required for oxidative stress tolerance |
| *SKN7* | 8200248 | Transcription factor |
| *MSN4* | 8198341 | Transcriptional activator related to Msn2p |
| *OPY2* | 8199837 | Transcription factor |
| *SWI6* | 8201343 | Transcription cofactor |
| *SWI6* | 8201343 | Transcription cofactor |

**Table S4 List of genes and primers for RT-qPCR used in this study**

| Gene name | Primer sequence (5’-3’) |
| --- | --- |
| *ACT1* | F: TATTGAAGTTGAAGCCCTCTGAGC  R: CCTTCCGTGTGCAAATGAAACAC |
| *CAT* | F: TTCGACAACGCTAATCACGCTAAC  R: TCACCTCAAACTCACCGAAAGCT |
| *MPV17* | F: AGAAATCCGATCATCACCAACGG  R: CTCCAATGGGCGCAAAGATGAT |
| *PMP47* | F: GGGATAGCAGTCAACAACTTCATTT  R: TACGGGAAATCACACCAGCAATAG |
| *DDO* | F: TCTCTGATTGATAAGGGGACTGGG  R: CGGTGCAAATTGGTGTAAGTAAACT |
| *XDH* | F: GGAGATCGACATGACTGAACTCTTC  R: GCTGAGCTTTTGTTGTAGGCTTG |

FIGURES


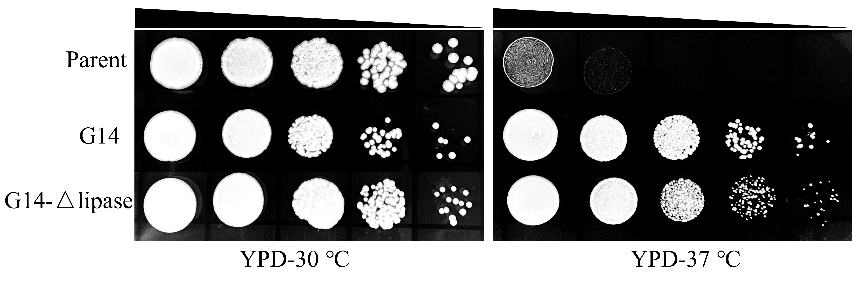


Fig. S1. **Spot assay to show the impact of lipase gene in the thermotolerance of G14.** The thermotolerance of G14 was still maintained after inactivation of lipase gene.


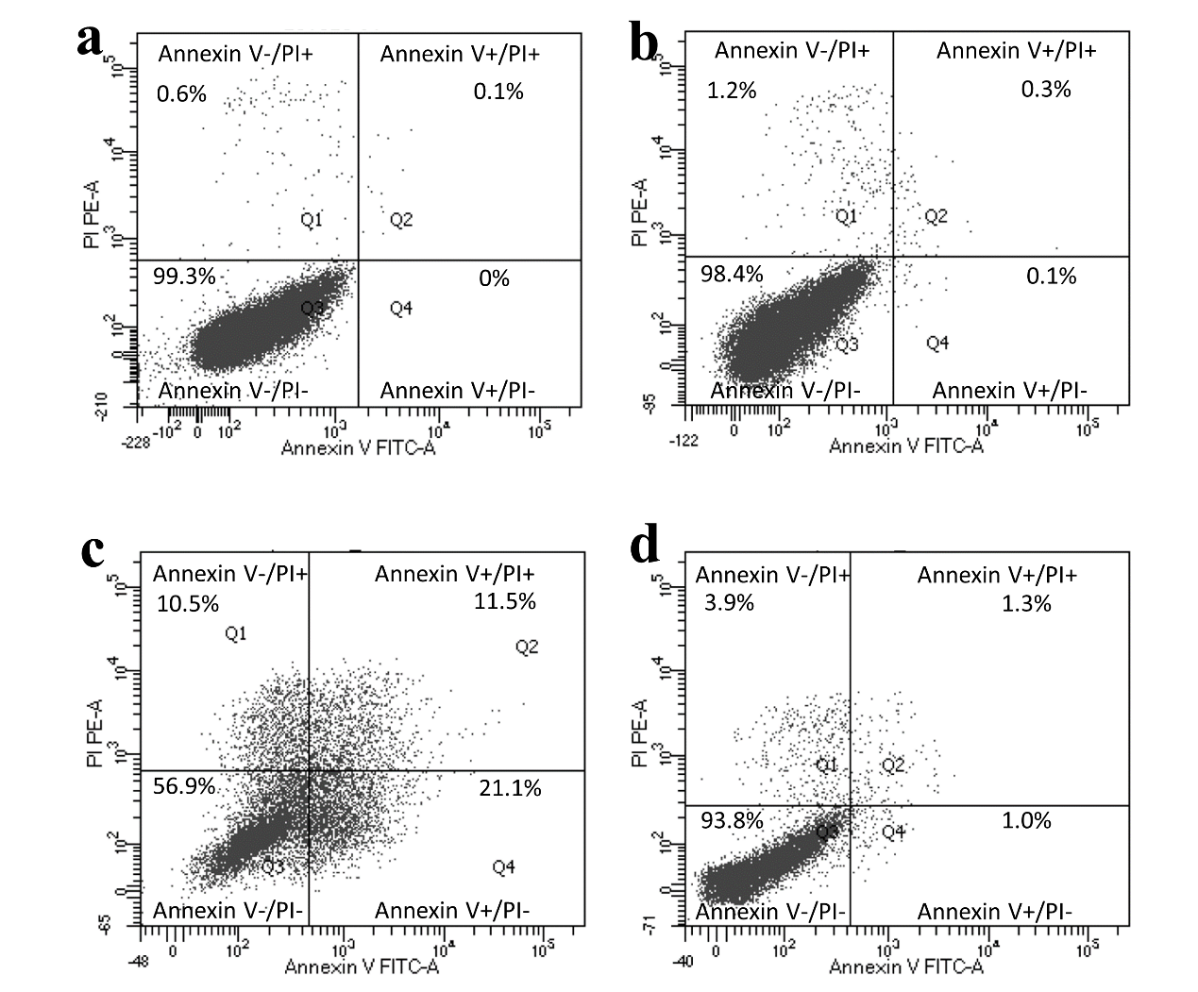


Fig. S2. **The** **Annexin V-FITC/PI staining was analyzed by flow cytometry for** **cell apoptosis and necrosis.** (a) The parent under non-stress condition (30 °C); (b) Thermotolerant strain G14 under non-stress condition (30 °C); (c) The parent under thermal stress (37 °C); (d) Thermotolerant strain G14 under thermal stress (37 °C). More than 10,000 events of each sample were test. Spots in Q3, Q4, Q2, and Q1 indicate that cells were alive, early apoptotic, late apoptotic (necrotic), and dead, respectively.


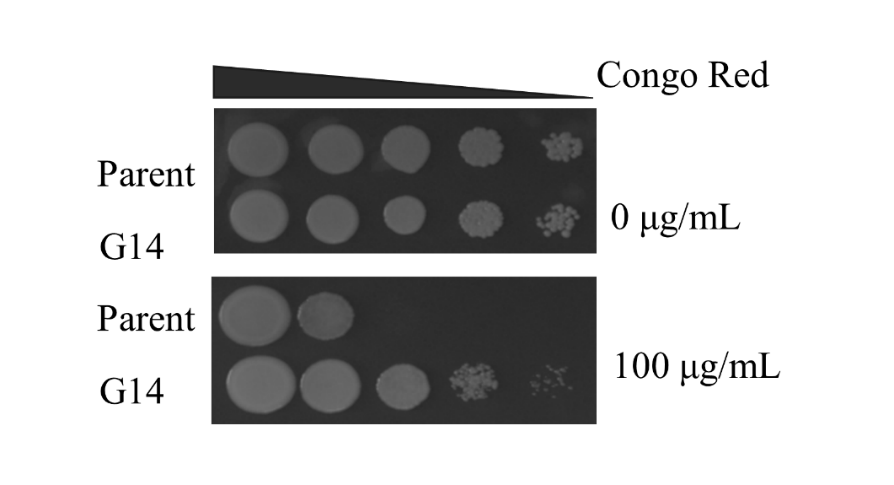


Fig. S3. **The test of cell wall integrity by** **Congo Red resistance test.**


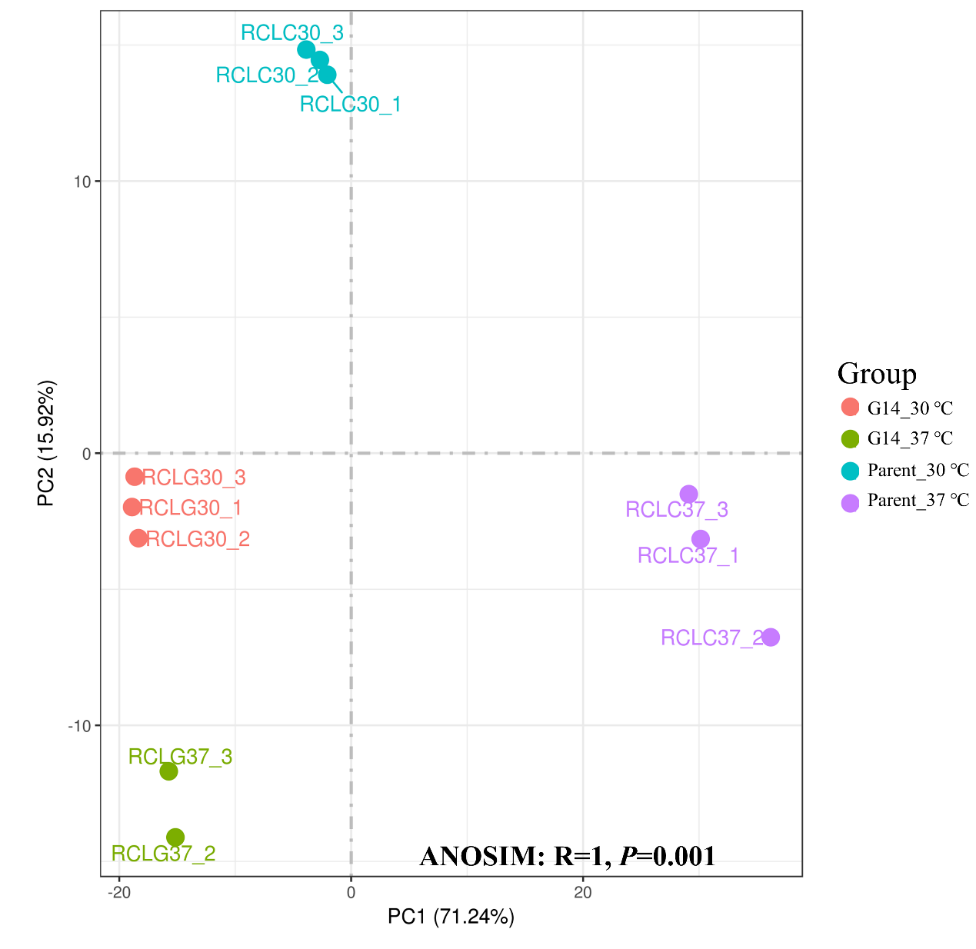


Fig. S4. **The correlation of samples by principal-component analysis (PCA) and analysis of similarities (ANOSIM).** PCA analysis showed good repeatability of each biological replicate within each group. The results of PCA and ANOSIM showed that the data of RNA-seq in each group was significantly different. The results indicated that the data could be employed for the following analysis.


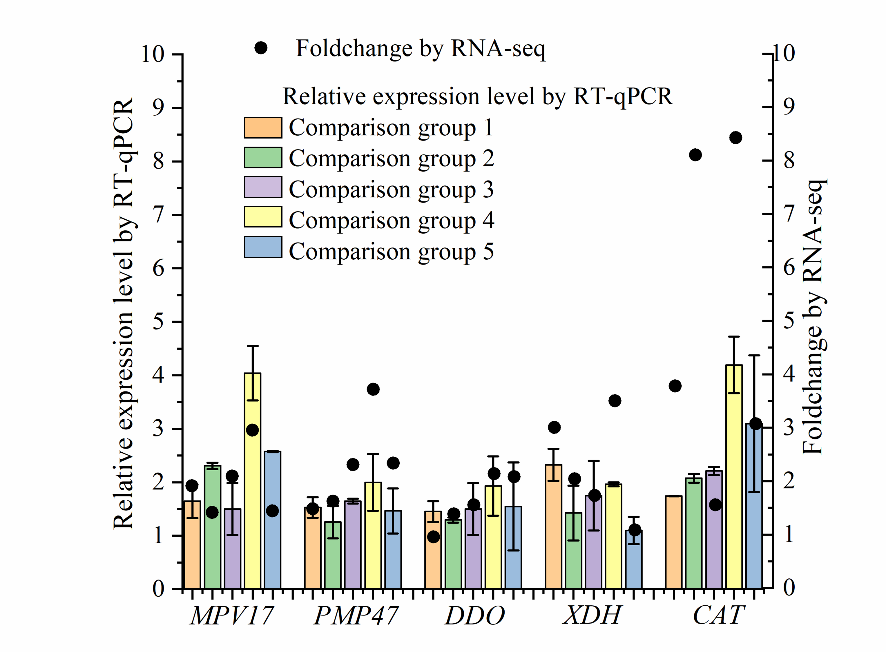


Fig. S5. **Expression level of peroxisomal protein encoding genes in five comparison groups.** Relative expression levels of genes were measured by RT-qPCR and analysed by the 2^-[delta][delta]Ct^ method. The RT-qPCR data were consistent with the results obtained from RNA-seq. Comparison group 1: the parent under 37 °C versus the parent under 30 °C. Comparison group 2: G14 under 37 °C versus G14 under 30 °C. Comparison group 3: G14 under 30 °C versus the parent under 30 °C. Comparison group 4: G14 under 37 °C versus the parent under 30 °C. Comparison group 5: G14 under 37 °C versus the parent under 37 °C.


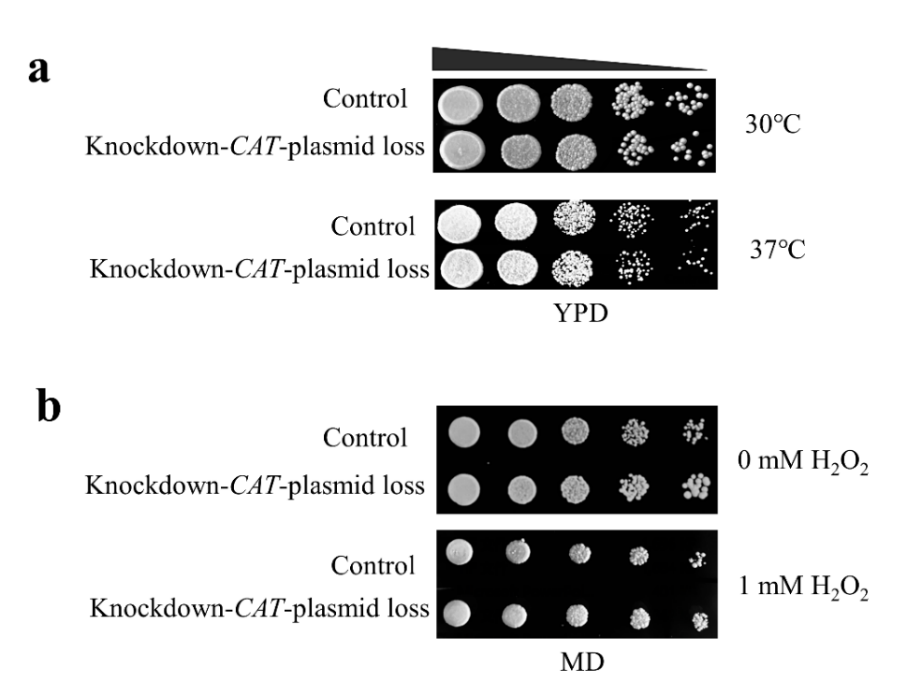


Fig. S6. **Spot assay to show the stress tolerance of G14 was recovered after losing *CAT* gene knockdown episomal plasmid.** (a) Thermotolerance test; (b) Oxidative stress tolerance test.


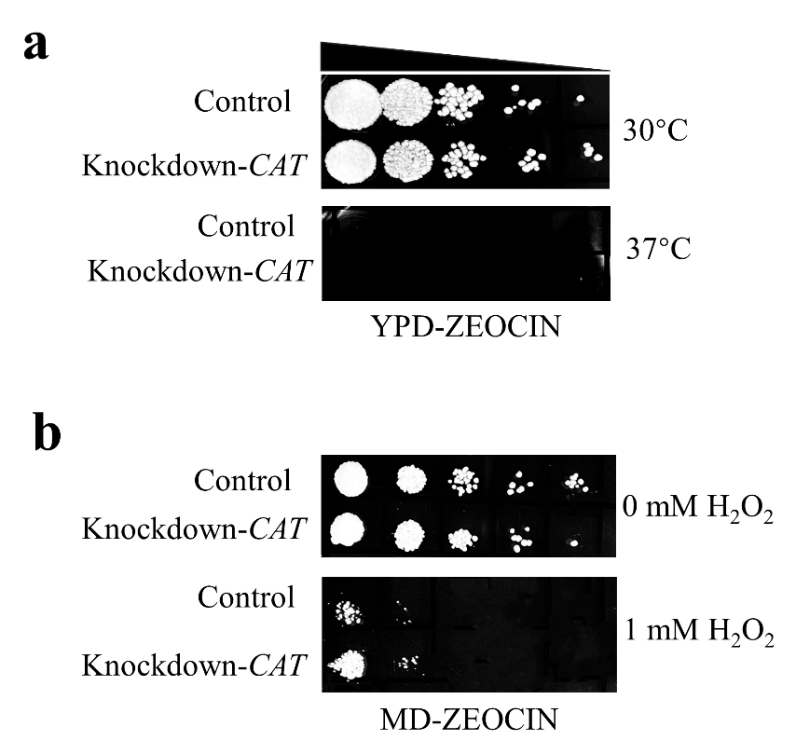


Fig. S7. **Spot assay** **to test the impact caused by gene knockdown of *CAT* on cell growth of the parent at different temperature and with/without H_2_O_2_.** (a) Thermotolerance test; (b) Oxidative stress tolerance test.

1. *Corresponding authors:

   E-mail address: yuxw@jiangnan.edu.cn (X.W. Yu). [↑](#footnote-ref-1)
